# Open data models for smart health interconnected applications: the example of openEHR

**DOI:** 10.1186/s12911-016-0376-2

**Published:** 2016-10-22

**Authors:** Hans Demski, Sebastian Garde, Claudia Hildebrand

**Affiliations:** 1 Helmholtz Zentrum München, Deutsches Forschungszentrum für Gesundheit und Umwelt (GmbH), Ingolstädter Landstraße 1, 85764 Neuherberg, Germany; 2 Ocean Informatics, 124 Cromwell Road, Kensington, London, SW7 4ET United Kingdom

**Keywords:** Clinical information models, Electronic health record, Model-driven engineering, Open data models, openEHR, Semantic interoperability

## Abstract

**Background:**

Smart Health is known as a concept that enhances networking, intelligent data processing and combining patient data with other parameters. Open data models can play an important role in creating a framework for providing interoperable data services that support the development of innovative Smart Health applications profiting from data fusion and sharing.

**Methods:**

This article describes a model-driven engineering approach based on standardized clinical information models and explores its application for the development of interoperable electronic health record systems. The following possible model-driven procedures were considered: provision of data schemes for data exchange, automated generation of artefacts for application development and native platforms that directly execute the models. The applicability of the approach in practice was examined using the openEHR framework as an example.

**Results:**

A comprehensive infrastructure for model-driven engineering of electronic health records is presented using the example of the openEHR framework. It is shown that data schema definitions to be used in common practice software development processes can be derived from domain models. The capabilities for automatic creation of implementation artefacts (e.g., data entry forms) are demonstrated. Complementary programming libraries and frameworks that foster the use of open data models are introduced. Several compatible health data platforms are listed. They provide standard based interfaces for interconnecting with further applications.

**Conclusion:**

Open data models help build a framework for interoperable data services that support the development of innovative Smart Health applications. Related tools for model-driven application development foster semantic interoperability and interconnected innovative applications.

## Background

The use of technologies for data acquisition, processing, and analysis of healthcare data is part of modern healthcare. Mobile applications as well as sensors enable consumers to collect, and share a multitude of measurements (e.g., blood glucose) and observations (e.g., food intake, activity levels) [1, 2]. Enhanced networking, intelligent data processing, and combining patient data with other parameters (e.g., environmental data) provide new opportunities for clinical research. This concept is known as Smart Health [3].

While there has been a dramatic rise in the volume of digital health data being recorded [4], a lot of data remains trapped in electronic health record (EHR) systems. The exchange of data between different information systems frequently proves difficult and is often not realized [5]. This is partly due to the great variety and large scale of biomedical concepts captured. New scientific discoveries and the constantly evolving medical practice require ongoing adaptations of medical information systems [6]; the growing demand on networking and automated evaluation call for data integration and semantic interoperability.

Standard based and common Open Data Models (ODMs) foster exchange, discussion and consensus regarding the data models in the medical domain [4]. ODMs that are made accessible to the public can provide syntactic and semantic specifications and meta-information about the shared data. They promote transparency and, at the same time, enable efficient data integration and reliable analysis within Smart Health systems. Existing clinical information models (CIMs) which are published as ODMs can be reused, They thus speed up the development of new applications and ease the maintenance of existing ones. This is in line with the model-driven software development methodology [7] which facilitates the handling of complex platforms by applying domain specific models. Standardization and transparency of data models used in healthcare information systems are key elements for data sharing. Further they are vital for a “smart” analysis of the emerging large scale data pools containing heterogeneous data from different sources.

Various standardization initiatives have been working on the definition of shareable CIMs. The Clinical Data Interchange Standards Consortium (CDISC) develops open standards to improve medical research and to ensure a link with healthcare [8] which is so far missing. The CDISC Operational Data Model, for example, specifies the information that needs to be shared among different software systems at three different stages: during study setup, operation, and analysis [9].

HL7 provides a set of standards for transferring clinical and administrative data between various healthcare providers [10]. Its primary standards are the Messaging Standard of Version 2, the Clinical Document Architecture (CDA), and the Continuity of Care Document (CCD). While the Messaging Standard provides an interoperability specification for health and medical transactions, CDA describes an exchange model for clinical documents, based on HL7 Version 3. CCD is a specification for the exchange of medical summaries, based on CDA. The recent FHIR draft standard [11] includes Resource definitions that represent granular Clinical Concepts and combines those with RESTful web services.

The openEHR foundation [12] publishes comprehensive open specifications for a flexible Electronic Health Record architecture. It started in the early 1990s, based on the results of the European Union’s GEHR-Project. Over the last few decades the specifications have been refined in many different projects. openEHR introduced a reference model and – on top of this – clinical models (so-called archetypes) for the definition of shareable CIMs. openEHR provides tools that make it possible to define archetype models in an international collaborative approach. The resulting shareable CIMs have been published in an online repository [13]. The international ISO 13606 [14] was developed on the basis of the openEHR approach for the purpose of electronic health information exchange.

The Clinical Information Modelling Initiative (CIMI) [15] aims to provide shared implementable clinical information models as logical models that represent structured and computable meta-models. This work is related to the recently published Technical Specification ISO/TS 13972 Detailed clinical models, characteristics and processes [16], that follow the modelling approach described in the ISO Health Informatics Profiling Framework [17].

### Objective

Due to the complexity of the healthcare domain and its constantly evolving practice, the deployment of interoperable EHR systems and related applications for Smart Health is challenging. The objective of this analysis is to explore to what extent ODMs can facilitate the implementation of EHR systems and interconnected applications. The model-driven engineering methodology is used for application development.

## Methods

Model-driven engineering is a software development methodology focusing on the creation and exploitation of domain models, which are conceptual models of a specific application field [7]. Standardized and jointly designed models support the development of software and systems. This approach aims to increase productivity by facilitating a system’s compatibility through the reuse of standardized models. In addition, it simplifies the design process and promotes communication between developers via standard terminology and the application of best practices in an application domain. Some prominent initiatives that follow this approach are the Model-driven Architecture (MDA) by the Object Management Group (OMG) and the Eclipse ecosystem of modelling tools (Eclipse Modelling Framework) [18, 19]. Triggered by our experiences with the application of the openEHR methodology within the EMPOWER project [20], where ODMs for diabetes patients were developed [21] and implemented within a web based personal health record and a complementary mobile application [22], it was explored how this universal method can be applied to support the development and implementation of interoperable EHR systems. The particular focus was on ODMs for specifying clinical data models and their usefulness for interconnected Smart Health applications.

The construction of systems can profit from ODMs in various ways; the level of integration and automatization of processes may vary. In this paper, the following three model-driven development (MDD) procedures were considered:

- provision of data schemes for data exchange
- data model related artefacts for application development
- platforms that are able to execute the models natively

It was analyzed whether ODMs can serve as a basis for implementing health information systems. The applicability of a model-driven framework in practice was examined using openEHR as an example. Therefore, the openEHR inventory of tools [23, 24] was searched for solutions that offer assets facilitating the implementation of EHR applications.

First the generation of data schemas based on this approach was investigated and the capabilities of existing modelling tools were explored. Next it was examined whether MDD based on ODMs can facilitate the construction of Smart Health systems considering specifically the supportive methods for the generation of implementation artefacts. Finally, we looked into available health data platforms and analysed their capabilities related to ODMs.

## Results

The applicability of a model-driven approach for EHR development was demonstrated by using openEHR as an example. To begin with, the comprehensive openEHR framework is being introduced.

### Model-driven engineering with ODMs: the example of openEHR

The Dual Model Approach followed by openEHR relies on ODMs that foster semantic interoperability and promise to deliver flexible systems for sustainable EHR data management. This was confirmed by Atalag et al. [25]. He reported a significant reduction of implementation time while increasing maintainability when he used this methodology for the development of clinical information systems. The infrastructure for development and maintenance of information models is in place; the openEHR Clinical Knowledge Manager (CKM) is an established international, online clinical knowledge resource. It supports the management of ODMs, including a review and publication process, and it provides governance which allows the development of CIMs -with the support of domain experts- in a public and collaborative approach. Several instances of the openEHR CKM are worldwide in use [26]. So far, the openEHR Foundation has made more than 500 clinical data models available in its online repository [27]. They are being reviewed by an international community and will be published in due course.

In our study the openEHR website [13] was searched for information regarding model-driven engineering tools. Existing assets, freely available tools as well as open source components, that support the implementation of applications based on openEHR ODMs are shown in Table 1.

**Table 1:** List of openEHR tools, frameworks and platforms

Asset	Type	Provider	Link
Archetype Editor	Modelling tool	Ocean Informatics/openEHR foundation	http://www.openehr.org/downloads/archetypeeditor/home
Template Designer	Modelling tool	Ocean Informatics/openEHR foundation	http://www.openehr.org/downloads/modellingtools
ADL Workbench	Modelling tool	Ocean Informatics/openEHR foundation	http://openehr.org/downloads/ADLworkbench/home
Clinical Knowledge Manager	Online repository	Ocean Informatics	http://www.openehr.org/ckm/
LinkEHR Editor	Modelling tool	Universitat Politècnica de València	http://linkehr.com/download.html
ADL Designer	Modelling tool	Marand	https://github.com/openEHR/adl-designer
GDL Tools	Guideline definition tool	Cambio Healthcare Systems	http://sourceforge.net/projects/gdl-editor/
Java Reference Implementation	Programming Library	openEHR	https://openehr.atlassian.net/wiki/display/projects/Java+Project+Download
adl2-core Reference Implementation	Programming Library	openEHR	https://github.com/openEHR/adl2-core/
openEHR.NET	Programming Library	Ocean Informatics	http://openehr.codeplex.com
GastrOS	EHR framework	University of Auckland	http://gastros.codeplex.com/
Think!EHR	Health data platform	Marand	http://www.marand-think.com/
MedRecord	Health data platform	MedVision	https://medrecord.nl/
Multiprac	Health data platform	Ocean Informatics	https://oceaninformatics.com/solutions/clinical_product_suite

The next chapters give some examples of applying the three MDD approaches.

### Data schemes for data exchange

**Fig. 1:**
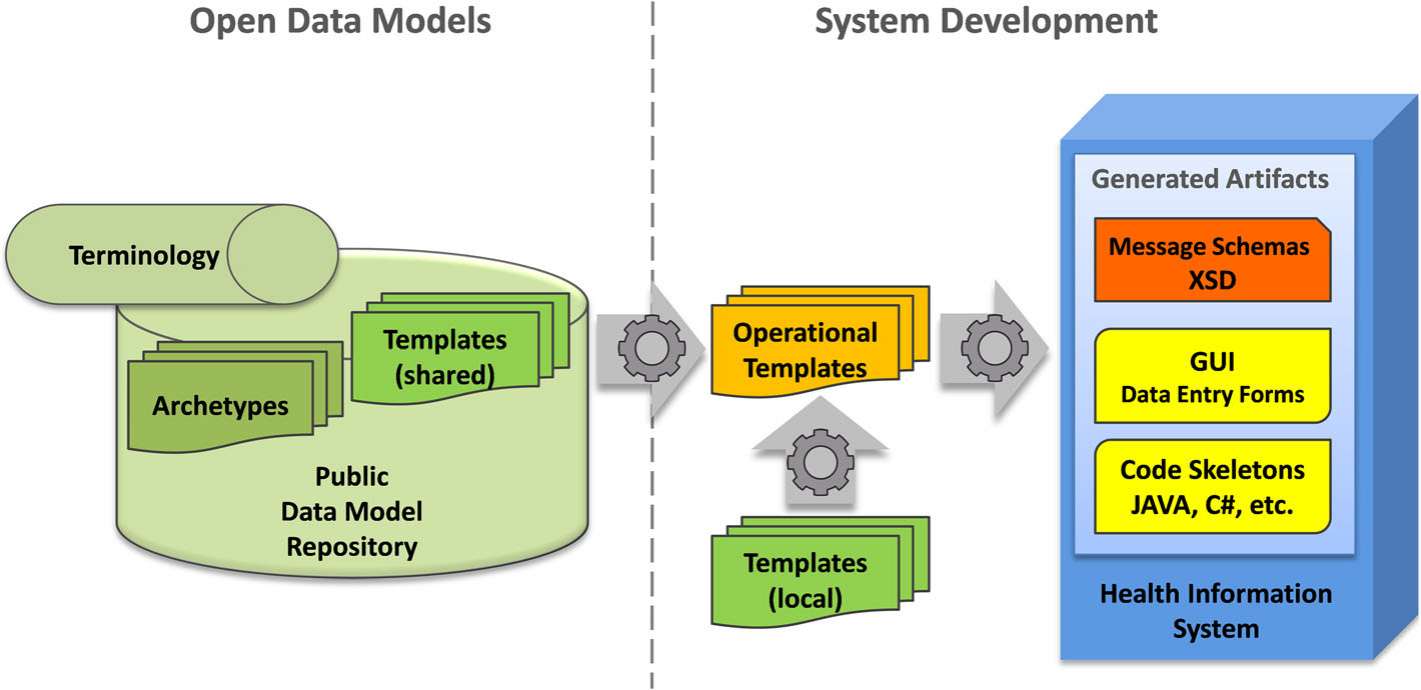
openEHR data models and artefacts supporting implementation

The first option for utilizing model-driven development is to have systems interact on a superficial layer only. Data schema definitions are derived from the ODMs in a common format to be applied for implementing data exchange interfaces. The translation of the specialized ODMs into, for example, XML Schema Definitions (XSD) makes it possible to apply the models without any need for extensive background knowledge. A special training in medical information modelling and terminologies is not required. This method facilitates semantic interoperable data exchange; there is no need for the complexity of the underlying CIMs to appear at the layer of interface implementation. The data that are captured on basis of the derived and simplified schemas are compliant with the underlying openEHR CIMs. They can be mapped to and transformed into the original data representation format. This way, legacy applications and systems which do not conform to the advanced ODMs can easily be integrated [28]. The CIMs can be incorporated by simply applying common practice software development processes (e.g., delivering XML data via a REST interface). The openEHR templates [29] support the adaptation of the archetype models to local requirements (Fig. 1). Thus, it is possible to combine archetypes, to mask elements that are not needed, to set units and to define value sets. The Template Designer can be used, even by non-experts, to customize the ODMs (e.g., openEHR archetypes and templates) for special needs. Amongst other possible usages for MDD the templates help to provide a suitable target for mapping to data schemes of individual systems, which is a core task within data integration processes.

The modelling tools listed in Table 1 allow the export of various artefacts that can be reused for application development, like XML Schema Definitions (XSD) and JavaScript Object Notation (JSON) templates. One example for a project that is successfully using this approach is the national health information exchange network in Slovenia. It defines clinical data models with the CKM [30] and has established an interoperability backbone based on Integrating the Healthcare Enterprise (IHE).

### Artefacts for application development

A more advanced alternative of application development is the use of model-driven engineering tools for automatic creation of implementation artefacts. This procedure delivers code skeletons for the application in software development (e.g., for data capturing, storage and retrieval). In order to produce the artefacts needed for a certain use case specific converters can be developed. If, for example, data entry forms for capturing information with a mobile application are needed, they can be built on basis of the ODMs by an automatic procedure that utilizes the information contained in the domain models [31].

**Fig. 2:**
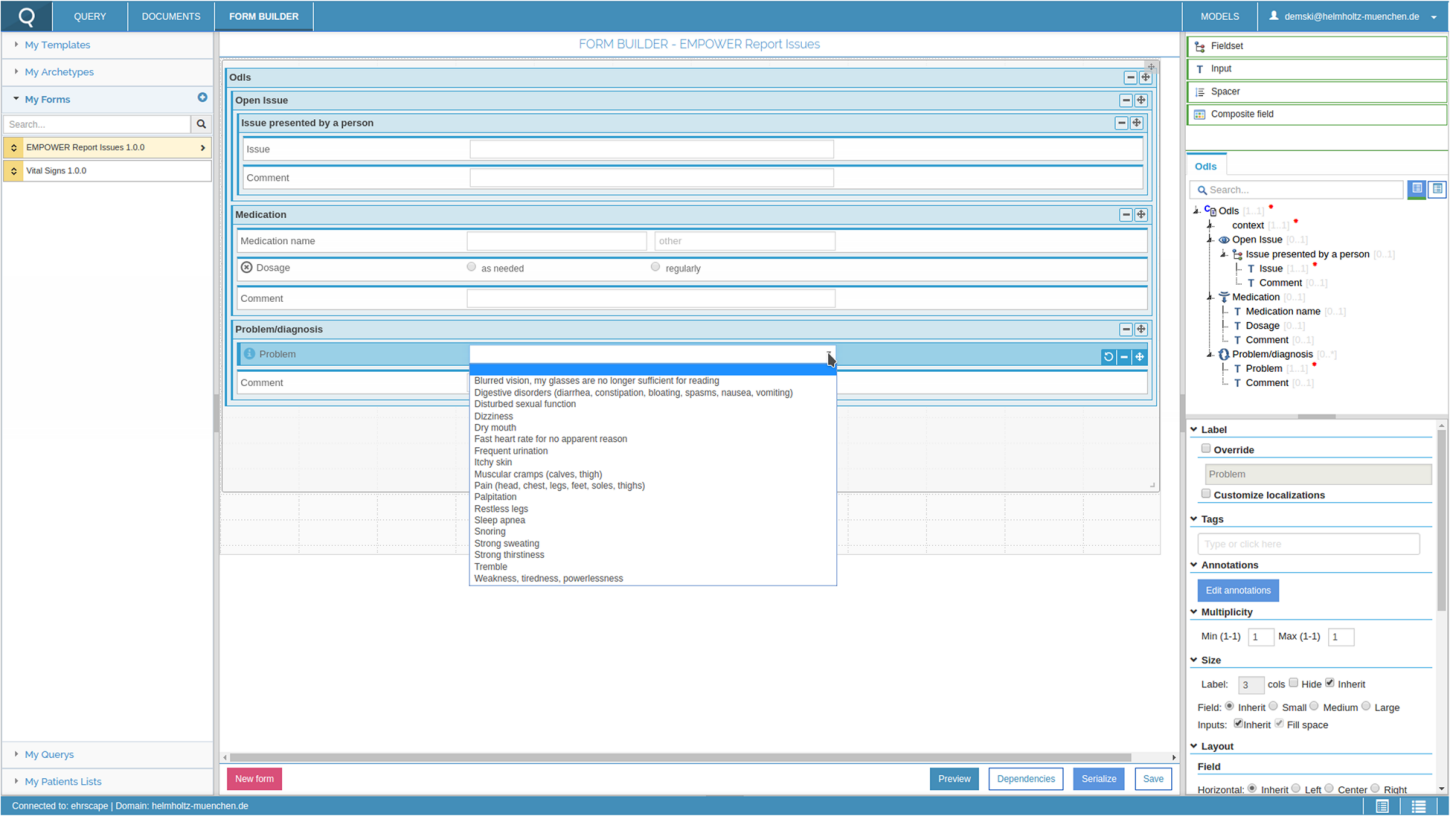
EHRscape Form Builder [52]

In openEHR, the Operational Template is a “flattened” XML-based representation of a template which provides a complete specification for a localized data model. Common openEHR tools like the Template Designer and the CKM can provide this as well as a couple of other formats for a given template and the corresponding archetypes. All archetype references are resolved and all the desired terminology sections are contained within a single file. This way all the relevant information is compiled into a single document, which contains only the language translations needed. Specialized tools can consume the Operational Templates and produce a myriad of downstream artefacts ranging from database schemas over generated source code to screen forms. Figure 2 shows one example of a screen form builder based on Operational Templates. This approach enables the development of specific transformationss that produce the artefacts needed for a certain use case. Similar approaches for generating entry forms based on openEHR CIMs were presented by Atalag [25], Chen [32] and Duftschmid [33].

The results of the model based artefact generation are ideally combined with existing programming libraries to foster application development. A JAVA and a.NET reference implementation of the openEHR reference and archetype model and other core semantics are available (Table 1). Various projects, for example a Ruby reference implementation, are working on open source components for openEHR [34]. Academic application frameworks explore the potential of ODMs within EHR systems. GastrOS [35], for example, developed an openEHR based endoscopic reporting application that is capable of dynamically generating the graphical user interfaces (GUI) from underlying domain knowledge models. EHRServer [36] provides a Service-oriented, REST based, openEHR repository for clinical data.

### Native EHR platforms

**Fig. 3:**
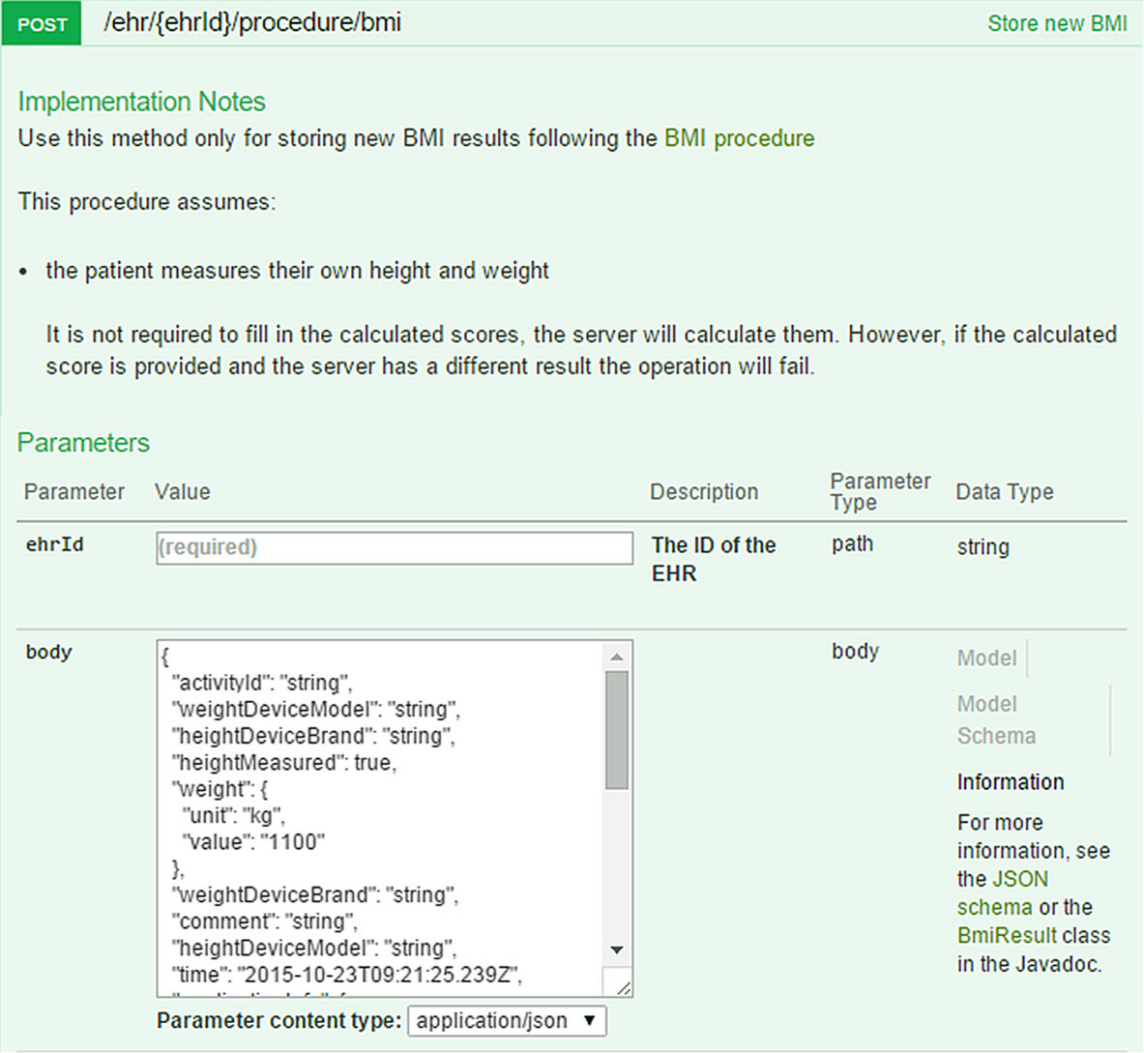
MedRecord documentation of the REST service for external applications [53]

Health data platforms provide the third MDD option natively supporting ODMs. They are ready for use. These health data platforms are complete EHR solutions. They enable the use of archetypes for defining clinical data models to be processed within the application software. Such frameworks provide tools that typically utilize ODMs for the definition of screen forms. They facilitate decision support and enable the user to formulate queries. Ideally, they provide standard interfaces (e.g., HL7 V2 messages, HL7 CDA) for data exchange and support common protocols like REST for interlinking with mobile applications (Fig. 3).

The openEHR specification for data querying (AQL) has been implemented in the Think!EHR platform, which provides an interactive AQL based query builder.

The eHealth Moscow project that developed a centralized EHR system used Marand Think!EHR platform [37], which is based on this approach. Also DIPS ASA, that is distributing an EHR for hospitals in Norway, is utilizing the Think!EHR platform. A further example is MedVision, that is developing mobile applications with care plan support based on its MedRecord platform [38]. Ocean Informatics has successfully established the LinkedEHR shared care plan, which is based on its MultiPrac platform, in the Western Sydney region in Australia [39].

**Fig. 4:**
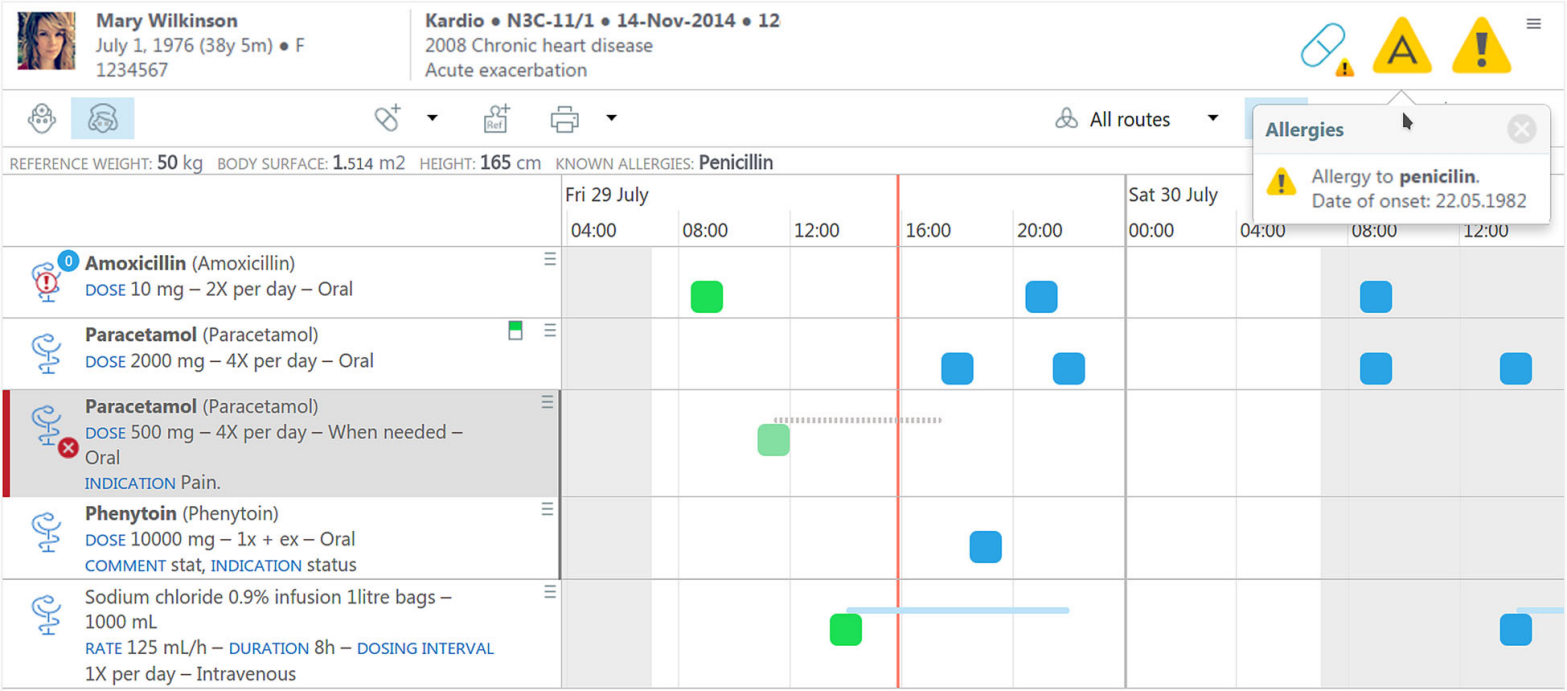
openEP Demonstrator for ePrescription [54]

Other implementations of openEHR existing worldwide confirm the applicability of the approach [40]. The fairly recently realized Code4Health [41] platform from NHS England provides a demonstrator for an open ecosystem for health applications and services. It includes an openEHR repository containing test data and exposes SMART [42] and FHIR APIs [11] in addition to the native openEHR service API. These common interfaces, together with the shared ODMs, facilitate the interconnection of a multitude of additional applications with different scopes for diverse use cases. The platform’s open architecture aims to support the parallel usage of applications from different suppliers. Several demonstration applications are in place showing the capabilities of the solution to plug in a great variety of specialized applications. The implementation of an existing product for ePrescription and its adaptation to the special local requirements has successfully served as proof of concept (Fig. 4).

## Discussion

The model-driven methods presented only show an excerpt of possible approaches for software development and the promotion of data integration in healthcare. Two other prominent activities are worth mentioning. HL7 FHIR combines a lean webservice application programming interface with a common core dataset that can easily be extended if needed. A Design Guide helps with the development of resources and extensions [43]; multiple implementation libraries and many examples are available that facilitate design and development [44]. Originally spreadsheets, in addition to UML class diagrams and XHTML files, were used for content definitions and their documentation [45]. Meanwhile the more sophisticated Forge toolset has been developed by Furore and is recommended by HL7 for FHIR data modelling [46]. Sundvall et al. introduced a REST based system architecture, which is similar to FHIR, for archetype-based EHR systems [47]. Another initiative promoting data integration in healthcare is the Clinical Data Interchange Standards Consortium (CDISC). It provides a comprehensive suite of vendor-neutral and platform-independent standards for clinical research data and metadata. In the BRIDG project [48] CDISC is collaborating with HL7 on the development of an overarching domain analysis model that will include the healthcare domain. This facilitates semantic interoperability between clinical research and the healthcare domain.

A myriad of other ODM initiatives, for example CIMI, exist. These can be used in the area of Smart Health for promoting interconnected applications in similar ways. The purpose of this study, however, was not to assess differences, advantages and disadvantages of individual ODMs in detail, but rather to assess the suitability of ODMs in supporting MDD in the area of Smart Health in general, using the example of one of the ODMs, namely openEHR. The openEHR approach has been proven to be successful in many open-source as well as commercial implementations. Proven deployments range from academic research institutions over non-profit and government organizations to public and private hospitals [49].

In addition, clinical data warehouses are becoming popular. These have to interconnect with a multitude of existing EHR systems and emerging mobile applications for healthcare that are developing at a fast pace. The depicted methodology has the potential to leverage ODMs for application to this purpose. The emerging field of self-monitoring data collected by patients themselves creates new opportunities for research but also better healthcare, provided that the challenges of integration and interpretation of these large and heterogeneous data pools are met.

The applicability of ODMs within a model-driven architecture for EHR development was demonstrated using openEHR as an example. openEHR realizes model-driven architecture with a comprehensive toolset for data modelling. In particular, it addresses the joint development of CIMs and the generation of implementation artefacts from models. MDD lowers the entry barrier for developing systems based on standardized ODMs for healthcare while at the same time speeding up application development. In addition, experts from the medical domain are empowered; they can participate in the definition of models without having to deal with the technical aspects of medical information systems. Smart Health systems which use model-driven engineering methodology in combination with ODMs are able to profit from the reuse of existing data models, from sustainable EHR data management and from data sharing in the best possible way.

It was depicted that standardized clinical ODMs can be used as a source for automatically generating data schemas that support established data formats for interoperable data exchange. The application of the derived common data schemas does not require any special know-how about CIMs. This fosters the development of innovative applications for sharing the complex and diverse data that are required for healthcare; hence “data-liquidity” is promoted. Nevertheless, data integration based on XML and XSD is limited and does not enable semantic interoperability. An additional ontological layer is needed, which, in our example, is provided by the openEHR archetype models. The data schemas are derived from underlying archetype models, that provide the rich metadata models, common elements and linkage to standard terminologies which are missing in an XML/XSD only approach [50]. Naturally, for true semantic interoperability it is necessary that the clinical models are harmonized across institutional and regional boundaries as well as across all health professions [51]. For openEHR, these efforts are supported by the CKM.

ISO/TS 13972 “Detailed clinical models, characteristics and processes” summarizes these benefits as follows: “standardization of clinical concept representation is a desirable and cost effective way to aggregate data from multiple health IT systems and operate as a cohesive whole” [16]. In order to reap the benefits of ODMs for Smart Health solutions, implementers are challenged to put the model-driven engineering methodology into practice. Three possible procedures, with a varying level of integration into systems development, were demonstrated in this paper.

## Conclusion

Using the example of openEHR it was shown that ODMs can support Smart Health in various ways: providing data schemes for data exchange, enabling automatic artefacts generation for application development and facilitating health data platforms.

ODMs can be regarded as enablers for interconnecting the fragmented and highly diverse stand-alone applications in healthcare.

## Abbreviations

ADL: Archetype definition language
AQL: Archetype querying language
CCD: Continuity of care document
CDA: Clinical document architecture
CDISC: Clinical data interchange standards consortium
CIMI: Clinical information modelling initiative
CIMs: Clinical information models
CKM: Clinical knowledge manager
EHR: Electronic health record
HL7: Health Level Seven International
IHE: Integrating the Healthcare Enterprise
ISO: International Organization for Standardization
MDD: Model-driven development
ODMs: Open data models
REST: Representational state transfer
XHTML: Extensible hypertext markup language
XML: Extensible markup language
